# Conservative Surgical Management and Delayed Primary Closure in Fournier’s Gangrene of Perianal Origin: A Case Report and Literature Review

**DOI:** 10.7759/cureus.116010

**Published:** 2026-09-09

**Authors:** Yunuén Maqueda Sánchez, Martin Jesus Mendez Bacelis, María Fernanda Frías Fraire

**Affiliations:** 1 Medicine, "Dr. Jesús Kumate Rodríguez" General Hospital of Cancún, Cancun, MEX; 2 General Medicine, Autonomous University of the State of Quintana Roo, Quintana Roo, MEX

**Keywords:** conservative surgical management, fournier gangrene, fournier’s gangrene, invasive surgical debridement, perineum reconstruction, secondary closure, type 2 diabetes mellitus (dm)

## Abstract

Fournier’s gangrene (FG) is a fulminant and life-threatening polymicrobial necrotizing fasciitis of the perineal, perianal, and genital regions. It typically affects patients with significant comorbidities, particularly poorly controlled diabetes mellitus, and is associated with high mortality rates. We describe the case of a 57-year-old male patient who presented with a one-month history of severe perineal pain, initially misdiagnosed as uncomplicated hemorrhoidal disease. Upon admission, the patient exhibited systemic inflammatory response syndrome and a large, purulent perianal mass. The patient underwent an emergency radical surgical debridement followed by three serial surgical washouts and strict glycemic control. Ultimately, successful delayed primary closure was achieved without the need for fecal diversion or complex reconstructive flaps. His postoperative course was favorable, with complete preservation of sphincter function. This report underscores the importance of considering early radical debridement and demonstrates that a staged conservative surgical approach can yield excellent functional and cosmetic outcomes in extensive perineo-scrotal defects.

## Introduction

Fournier’s gangrene (FG) is a rare, rapidly progressive, and life-threatening polymicrobial necrotizing fasciitis affecting the perineal, perianal, and urogenital regions, with an estimated global incidence of approximately 1.6 cases per 100,000 males annually and a strong male predominance (10:1) [1]. Originally described as an idiopathic condition in healthy young men, modern clinical understanding recognizes FG as a secondary manifestation of identifiable anorectal (30-50%), urogenital, or cutaneous infectious sources [2]. Microbiologically, it is predominantly polymicrobial, driven by synergistic interactions between aerobic organisms (e.g., Escherichia coli, Klebsiella pneumoniae, Pseudomonas aeruginosa) and anaerobes (e.g., Bacteroides fragilis, Clostridium spp.) [3]. Epidemiologically, type 2 diabetes mellitus represents the single most significant predisposing factor, identified in up to 60% of cases [3].

The hallmark of FG pathophysiology is an infectious endarteritis obliterans that induces local microvascular thrombosis, tissue hypoxia, and rapid liquefaction of fascial layers [4]. Early imaging, such as point-of-care ultrasound or non-contrast computed tomography (CT), can assist in ambiguous cases by identifying subcutaneous gas or deep fascial fluid collections [5]. However, cross-sectional imaging should never delay definitive surgical intervention when overt clinical signs of necrotizing infection are present [5]. Prompt initiation of broad-spectrum empirical intravenous antimicrobial therapy (covering Gram-positive, Gram-negative, and anaerobic pathogens) alongside rapid fluid resuscitation is essential [5]. Despite these measures, mortality rates remain high (20-40%) due to rapid fascial dissemination [6]. Complete and immediate surgical excision of necrotic tissue remains the unequivocal mainstay of treatment [6].

Following initial radical debridement, managing the residual soft tissue defect presents a critical reconstructive challenge [7]. While complex flap reconstruction or split-thickness skin grafting is often advocated, serial surgical washouts combined with delayed primary closure (DPC) offer a tissue-sparing approach that minimizes donor-site morbidity and operative complexity [7]. This report aims to demonstrate the feasibility, surgical protocol, and favorable functional outcomes of staged conservative debridement followed by DPC in extensive perianal FG without requiring fecal diversion or complex reconstructive flaps.

## Case presentation

A 57-year-old male patient presented with a one-month history of severe, burning perineal pain that gradually increased in intensity. He was initially managed in an outpatient setting for presumed hemorrhoidal disease with oral antibiotics, showing no clinical response. Two weeks prior to admission, the lesion expanded rapidly, accompanied by severe localized swelling, marked impairment in sitting, and spontaneous foul-smelling purulent discharge. His medical history was notable for poorly controlled type 2 diabetes mellitus, chronic anemia under active evaluation, and unintentional weight loss of approximately 10 kg over two months.

On physical examination, the patient was in moderate clinical distress. Baseline vital signs revealed a blood pressure of 110/70 mmHg (reference range: 90/60-120/80 mmHg), heart rate of 115 bpm (reference range: 60-100 bpm), respiratory rate of 22 breaths/min (reference range: 12-20 breaths/min), body temperature of 38.2 °C (reference range: 36.5-37.5 °C), and oxygen saturation of 96% on room air (reference range: 95-100%). Abdominal examination was soft, non-tender, without peritoneal signs. Regional examination of the perineum revealed marked induration, hyperthermia, purplish erythema, and asymmetrical enlargement of the right perineal region extending to the posterior scrotum. Digital rectal examination demonstrated preserved baseline anal sphincter tone, absence of endoluminal masses, and severe tenderness along the right posterior anorectal wall. A fistulous tract near the anal verge actively discharged liquefactive slough. Given the overt clinical signs of necrotizing soft tissue infection accompanied by systemic inflammatory response syndrome (SIRS), emergency surgical intervention was prioritized immediately; preoperative cross-sectional imaging (CT scan) was intentionally omitted to avoid dangerous delays in surgical source control.

Laboratory findings are summarized in Table 1.

**Table 1:** Laboratory findings on admission

Parameter	Result	Reference Range
White blood cell count	22,900/µL	4,000–11,000/µL
Neutrophils	70.0%	40–75%
Hemoglobin	10.2 g/dL	13.8–17.2 g/dL
Hematocrit	31.8%	41–50%
Platelet count	585,000 /µL	150,000–450,000 /µL
Serum Glucose	239 mg/dL	70–100 mg/dL
Blood Urea Nitrogen	38 mg/dL	7–20 mg/dL
Creatinine	0.9 mg/dL	0.7–1.3 mg/dL

Results showed severe leukocytosis (22,900/µL) with neutrophilia (70%) and marked hyperglycemia (239 mg/dL), consistent with systemic inflammation and acute glycemic decompensation.

The patient was immediately admitted for emergency surgery, aggressive fluid resuscitation, continuous regular insulin infusion, and empiric broad-spectrum coverage with intravenous meropenem (1 g IV every 8 hours) and vancomycin (1 g IV every 12 hours). Initial intraoperative wound cultures subsequently confirmed a polymicrobial growth featuring Escherichia coli and Streptococcus anginosus. Antimicrobial coverage was continued for 14 days until clinical signs and systemic inflammatory markers normalized.

The surgical protocol consisted of staged interventions, summarized in Table 2.

**Table 2:** Surgical protocol and clinical timeline of staged interventions

Postoperative Day	Surgical Intervention	Intraoperative Findings & Outcome
Day 0	Emergency radical surgical debridement	Extensive subcutaneous liquefactive necrosis; ~100 mL purulent exudate evacuated
Day 4	Second surgical washout	High-pressure saline irrigation and sharp removal of residual fibrinous slough
Day 16	Third surgical washout	Clean surgical margins, progressive cavity depth reduction, robust granulation
Day 28	Delayed primary closure (DPC)	Interrupted non-absorbable monofilament sutures placed without tension
Day 34	Hospital discharge	Complete wound apposition, optimal glycemic control, preserved continent functions

On day zero, the patient underwent emergency radical debridement of the right perineal region extending to the scrotal base. Intraoperative findings confirmed extensive liquefactive necrosis of subcutaneous tissue and fascia with approximately 100 mL of foul-smelling purulent fluid evacuated (Figure 1A).

**Figure 1:**
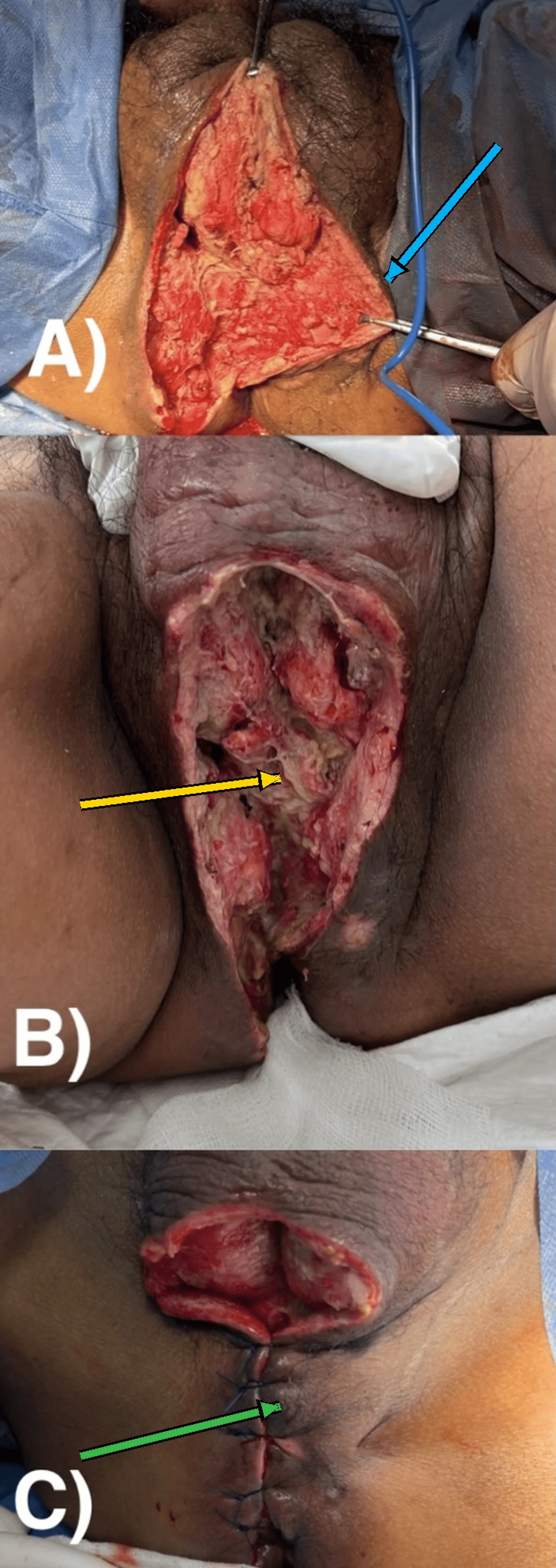
Serial surgical management and delayed primary closure of perineo-scrotal Fournier's gangrene (A) Initial radical surgical debridement on day zero demonstrating the extensive soft tissue defect and resected fascial margins (blue arrow); (B) Postoperative day 16 showing clean surgical margins and a robust granulating tissue bed (yellow arrow) following serial high-pressure washouts; (C) Postoperative day 28 demonstrating successful delayed primary closure with non-absorbable monofilament sutures placed without tension along the perineal midline (green arrow).

On postoperative day four, a second surgical washout with high-pressure saline irrigation and sharp debridement of residual fibrinous slough was performed. By day 16, a third surgical washout demonstrated clean surgical margins, progressive cavity depth reduction, and robust red granulation tissue (Figure 1B).

On day 28, following significant spontaneous wound contraction and verification of clean granulating tissue without persistent bioburden, the patient underwent a fourth surgical intervention for delayed primary closure. Full edge apposition was successfully achieved using interrupted non-absorbable monofilament sutures (2-0 polypropylene) placed without tension (Figure 1C).

## Discussion

FG remains a severe surgical emergency with reported mortality rates ranging between 20% and 40%, primarily driven by delayed presentation and rapid fascial dissemination [1]. Early clinical features are frequently insidious, particularly in diabetic patients where autonomic neuropathy can obscure early physical findings [2]. As observed in our patient, initial outpatient management for presumed benign anorectal conditions represents a common diagnostic pitfall documented throughout the literature [3]. Pathophysiologically, progressive microvascular thrombosis leads to extensive liquefactive tissue necrosis [4]. Prompt recognition of systemic toxicity combined with clinical or radiological evaluation ensures early surgical referral [5].

Aggressive radical debridement remains the mandatory cornerstone for infection control and mortality reduction [6]. However, managing the resulting extensive soft tissue defect presents a major reconstructive challenge [7]. While initial misdiagnosis remains frequent [8], traditional paradigms often advocate early definitive coverage via split-thickness skin grafts or regional advancement flaps [9]. Comparative analyses demonstrate that complex tissue transfer carries notable drawbacks, including extended operative times, donor-site morbidity, risk of flap necrosis, and substantial financial costs [9].

Conversely, serial high-pressure saline washouts combined with DPC leverage the high compliance, rich vascularity, and elastic properties of perineal and scrotal skin [9]. Serial washouts effectively clear fibrinous exudate and reduce bioburden, promoting robust granulation tissue [6,7]. In our case, staged washouts achieved sufficient wound contraction by day 28 to permit tension-free primary apposition. This conservative approach achieved full functional preservation, including complete anal sphincter continence, and optimal cosmesis without the surgical burden of regional flap reconstruction or the morbidity of a fecal diversion stoma [9].

## Conclusions

DPC following serial high-pressure surgical washouts represents a feasible, tissue-sparing, and safe reconstructive strategy for extensive perineo-scrotal defects secondary to FG in appropriately selected patients. By utilizing local tissue compliance and spontaneous wound contraction after complete infection control, this conservative approach can achieve favorable functional and cosmetic outcomes. Furthermore, it effectively avoids the operative complexity, donor-site morbidity, and prolonged surgical times associated with complex flap reconstruction or routine fecal diversion. Early recognition of necrotizing infection paired with prompt radical debridement remains essential to ensure patient survival prior to initiating staged reconstructive procedures. While these findings demonstrate excellent sphincter preservation and structural recovery in this specific presentation, clinical judgment must guide candidate selection based on defect extent and systemic stability. Overall, this staged management protocol serves as a viable and highly practical alternative in resource-conscious surgical settings.
